# Innovative Characterization Based on Stress Relaxation and Creep to Reveal the Tenderizing Effect of Ultrasound on Wooden Breast

**DOI:** 10.3390/foods10010195

**Published:** 2021-01-19

**Authors:** Zhen Li, Zongyun Yang, Yulong Zhang, Tong Lu, Xiaoqian Zhang, Yue Qi, Peng Wang, Xinglian Xu

**Affiliations:** Key Laboratory of Meat Processing and Quality Control, MOE, College of Food Science and Technology, Nanjing Agricultural University, Nanjing 210095, China; 2018808149@njau.edu.cn (Z.L.); 2018108052@njau.edu.cn (Z.Y.); 2017208012@njau.edu.cn (Y.Z.); 2019808143@njau.edu.cn (T.L.); 2019808110@njau.edu.cn (X.Z.); 2019108048@njau.edu.cn (Y.Q.); xlxus@njau.edu.cn (X.X.)

**Keywords:** ultrasound, stress relaxation, creep, tenderness, wooden breast

## Abstract

In order to explore a new strategy to characterize the texture of raw meat, based on the ultrasonic tenderized wooden breast (WB), this study proposed stress relaxation and creep to determine the rheological properties. Results showed that hardness was significantly decreased from 3625.61 g to 2643.64 g, and elasticity increased, after 600 W ultrasound treatment at 20 kHz for 20 min (on-time 2 s and off-time 3 s) at 4 °C. In addition, based on the transformation of creep data, a new indicator, slope ε′(t), was innovatively used to simulate a sensory feedback of hardness from the touch sensation, proving WB became tender at 600 W treatment due to the feedback speed to external force. These above results were confirmed by the reduced shear force, increased myofibril fragmentation index (MFI), decreased particle size, and increased myofibrillar protein degradation. Histology analysis and collagen suggested the tenderizing results was caused by muscle fiber rather than connective tissue. Overall, stress relaxation and creep had a potential to predict meat texture characteristics and 600 W ultrasound treatment was an effective strategy to reduce economic losses of WB.

## 1. Introduction

Consumers’ demand for healthy and high-quality products is one of the driving forces for the development of the food industry. Improving quality and efficiency through technological innovation has become a research hotspot. Thus, ultrasound as a “Green Food Processing” technology has been used to produce high-quality food [1].

The common indicators for evaluating meat tenderness include shear force [2], myofibril fragmentation index (MFI) [3], particle size [4] and texture profile analysis (TPA) [5]. The TPA is also known as the two-bite test. It mainly simulates the chewing movement of the human mouth and compresses the sample twice to obtain parameters such as hardness and springiness [6]. Obviously, TPA may not deliver the detailed texture information of raw meat. Therefore, an alternative method of TPA should be explored to obtain the texture parameters of raw meat.

The biochemical properties, chemical composition and cellular structure of a material determine its response to physical and mechanical testing [7]. The physical properties of food materials are related to sensory properties such as hardness, softness and brittleness [8]. Thus, consumers can recognize food quality through these characteristics. However, two important rheological indicators in raw meat, that is, stress relaxation and creep, have not been taken seriously. Compression tests are a golden standard for analyzing food texture [9]. The stress relaxation test is a typical compression method for studying the rheological properties of materials [10]. It mainly studies the law of the change in internal stress with time, which can reflect the internal structure and viscoelastic state of meat. Creep is a phenomenon by compression in which a certain force is applied to meat, and the deformation of meat gradually increases with time [11]. Stress relaxation and creep have the potential to predict meat texture characteristics and taste.

In 2014, Sihvo [12] described a kind of broiler breast as WB for the first time. WB myopathy is caused by a widespread fibrotic injury [13]. WB is hardened to the touch, and a hardened “ridge” can be observed on the caudal tip [14]. Histology observation showed the main characteristics of WB as necrosis and fibrosis, connective tissue infiltration, lipid infiltration, fiber diameter increase, fiber degeneration and regeneration, and inflammatory cell infiltration [12,15,16]. Analysis of meat quality found that shear force and compression force in WB are significantly higher than those in the normal breast (NB) [14,17], thus causing low consumer acceptance and economic loss. Xing [18] surveyed the incidence of WB in China, and the proportion was approximately 61.9%. Kuttappan [16] estimated that WB caused approximately $200 million economic losses due to trimming, drip loss, cook loss, downgrading and discarding. It is worth studying how to reduce the economic loss caused by WB.

Physical tenderization methods that have been widely studied include pulsed electric field [19], high pressure [20] and ultrasound [21]. Ultrasound is acoustic energy produced by waves with a vibration frequency that cannot be detected by humans [22]. The ultrasound process could transform electrical energy to vibrational energy and generate cavitation to produce chemical, physical or biological effects [21]. In meat processing, ultrasound was initially applied to determine fat and muscle of living cattle around the 1950s [23], and now it is a modern, novel, non-destructive, and cost-effective technique that is used to improve meat quality [24].

Therefore, this study applies ultrasound on WB as an example to (a) analyze raw meat texture by using stress relaxation and creep, (b) explore the best ultrasonic parameters, (c) determine the ultrasonic tenderization mechanism of WB, and (d) give inspiration to other tenderization methods. Through these, it can not only deepen the understanding of the tenderization mechanism through stress relaxation and creep, but also reduce the economic loss caused by WB.

## 2. Materials and Methods

### 2.1. Materials

Samples of one bird were judged as moderate WB only if both right and left breasts of this bird met with the moderate WB criterion (fillets that were hard throughout but flexible in the mid to caudal region) [25]. A total of 450 right breasts with moderate WB characteristics were selected to conduct our following research. After collection, breast samples were packed and placed in ice and transported to the laboratory. The cranial region of WB was trimmed off to remove all surface connective tissue and adipose. The cranial region was cut into 52 ± 2 g small cube (5 cm × 4 cm × 3 cm), and each cube was packed in individual bags. On 12 h postmortem, cubes were placed into a −20 °C refrigerator to be used up in 30 days. All cubes were thawed (24 h, 4 °C) before sonication. Analytical grade chemicals and reagents were used in this research.

### 2.2. Sample Preparation

**Figure 1:**
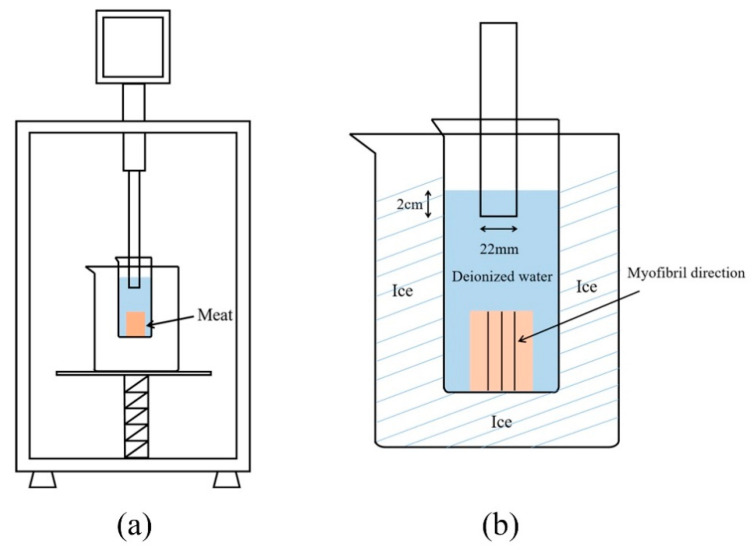
The schematic diagram of the ultrasound process, (**a**): Front view of ultrasound process; (**b**): The front detail of the ultrasound process.

The cubes immersed in 4 °C ultrapure water for 20 min without ultrasonic treatment were used as the control group (Control). Other samples were treated at 300, 600, 900, and 1200 W with a 20 kHz ultrasonic processor (YM-1500Y, Shanghai Yuming Instrument Co., Ltd., Shanghai, China) in 4 °C ultrapure water for 20 min (2 s on-time and 3 s off-time). During sonication, the 22 mm ultrasonic probe was inserted 2 cm into the water surface, and the beaker was wrapped in ice. The schematic diagram and detail of the ultrasound process are shown in Figure 1. After each treatment, the samples were immediately stored in ice for further use.

### 2.3. Shear Force

A C-LM3B tenderization analyzer (Northeast Agricultural University, Harbin, China) was used to conduct shear force tests according the method of Xing [18]. Cubes were packaged in plastic bags individually and then heated in 80 °C water bath until the core temperature reached 75 °C. After the samples were cooked, they were rinsed with running water to room temperature (25 °C) and wiped dry. Each cooked cube was cut into 3 cm × 1 cm × 1 cm slices parallel to the muscle fibers for the tests. The shear force (N) shown in the digital display when the slices were cut perpendicular to the muscle fibers was recorded. Each slice was cut two times, and 30 samples were used per treatment.

### 2.4. Stress Relaxation and Creep 

Before tests were conducted, the linear viscoelastic regime was determined by loading different strain and stress. The stress relaxation and creep of raw WB were determined through a texture analyzer (XT Plus, Stable Micro Systems Ltd., Godalming, UK) using a P50 probe. The cubes were cut to 2.5 cm × 2 cm × 2 cm. For stress relaxation tests, the compression strain was 30%; for creep, the compression force was 200 g. Other parameters were set as follows: 3.00 mm/s pre-test speed, 1.00 mm/s test speed, 3.00 mm/s post-test speed, 60 s time, and 5.0 g trigger force. 

### 2.5. Myofibril Fragmentation Index (MFI)

MFI was measured with reference to Hopkins [26] with some modifications. Exactly 0.3 g of raw meat was weighed and added with 4.5 mL of MFI buffer (0.1 M KCl, 7 mM KH_2_PO_4_, 18 mM K_2_HPO_4_, 1 mM EDTA·2Na, pH 7.0, 4 °C). The mixture was homogenized (Precellys Evolution Super Homogenizer, Bertin Technologies, Montigny-le-Bretonneux, France) twice at 10,000 rpm, 20 s each time. Then, the homogenate was filtered through two layers of medical gauze and centrifuged (Avanti J-E, Beckman Coulter, Inc., Brea, CA, USA) at 1000× *g* and 4 °C for 10 min. The centrifugal pellet was collected and added with 5 mL of MFI buffer and centrifuged twice. The precipitate was dissolved with 3 mL of buffer, and the myofibrillar protein (MP) solution for MFI was obtained and measured using biuret method with BSA as the standard protein. The protein concentration was adjusted to 0.5 mg/mL. The absorbance at 540 nm was determined immediately. MFI values were obtained by multiplying the absorbance at 540 nm by 200. 

### 2.6. Particle Size of MP

MP from WB was extracted according to Han [27] with some modifications. The raw WB cubes were minced in a precooled mincer (Knife Mill Grindomix GM 200, Retsch, Haan, Germany) at 3500 rpm for 30 s. Then, four times the volume ratio of standard salt solution (SSS) (0.1 M KCl, 20 mM K_2_HPO_4_, 20 mM KH_2_PO_4_, 1 mM EGTA, 2 mM MgCl_2_·6H_2_O, pH 7.0, 4 °C) was added to the meat batters and homogenized at 7000 rpm for 30 s in an ice bath. Afterwards, two layers of medical gauze were used to filter impurities, and the samples were centrifuged again at 2000× *g* for 10 min at 4 °C. The supernatant was discarded, and centrifugation was repeated three times. Thereafter, 0.5% Triton X-100 was added to SSS. Next, the precipitate was dissolved with four times the volume of 0.1 M KCl solution and homogenized in an ice bath at 7000 rpm for 30 s. Thereafter, the mixture was centrifuged at 2500× *g* for 10 min at 4 °C, and this process was repeated two times. Finally, the centrifugation product was MP, which was dissolved in PBS (0.6 M NaCl, 20 mM K_2_HPO_4_, 20 mM KH_2_PO_4_), and the concentrations were measured by biuret method with BSA as the standard protein.

The concentrations of MP solutions were adjusted to 1 mg/mL with PBS. The mean diameters of MP solution were analyzed using a Mastersizer 2000 instrument (Malvern Instruments Ltd., Worcestershire, UK).

### 2.7. Collagen Content

Total collagen was determined by measuring hydroxyproline concentration following the methods of Bergman and Loxley [28], Hill [29], Chang [30] and Vasanthi et al. [31] with minor modifications. Exactly 5 g of raw meat was weighed and added with 20 mL of 3 M H_2_SO_4_ solution in a glass tube, heated, and hydrolyzed in a drying oven (DGG-9240A, Shanghai Senxin Instrument Co., Ltd., Shanghai, China) at 110 °C for 18 h. After hydrolysis was completed, filter paper and active carbon were used to filter and decolor the hydrolysates. The filter paper and activated carbon were washed three times with ultrapure water. The collected water was mixed with the filtrate and diluted until 200 mL. Then, 20 mL of diluted hydrolysate was obtained, and pH was adjusted to 8.0 using 10 M and 1 M NaOH solutions. The hydrolysate was diluted to 250 mL with ultrapure water. Then, 4 mL of the hydrolysate was obtained to determine the hydroxyproline content. The total collagen content was equal to the hydroxyproline content multiplied by the factor 7.25 [32] and presented through percentage of the 5 g of meat.

The scheme of collagen solubility was modified from the methods of Fang et al. [33] and Hill [29] with minor modifications. The 5 g of raw meat sample was placed into a 50 mL centrifuge tube and added with 15 mL of 1/4 Ringer’s solution (1.8 g NaCl, 0.25 g KCl, 0.06 g CaCl_2_·6H_2_O, 0.05 g NaHCO_3_, 0.186 g iodoacetic acid) and homogenized (PD500, Prima Technology Group Co., Ltd., London, UK) at 7000 rpm for 30 s. The mixture was held in 77 °C water bath for 60 min and naturally cooled to room temperature before centrifuged for 20 min at 3300× *g*. Collecting the supernatant, and 8 mL of 1/4 Ringer’s solution was added to the precipitate and centrifuged again for 10 min at 3300× *g*. The precipitate was washed, and the washing liquid was collected. The supernatant from the two rounds of centrifugation was collected. Hydrolysis and calculation were carried out according to the method of measuring total collagen. 

### 2.8. Sodium Dodecyl Sulfate-Polyacrylamide Gel Electrophoresis (SDS-PAGE)

SDS-PAGE was performed following the method of Li [34] with some modifications. Briefly, 1 mg/mL of MP samples (30 μL) from 2.6 was mixed with 10 μL 4 × DTT SDS-PAGE loading buffer. Then, the mixture was heated at 100 °C for 10 min. The loading mixture (10 μL) and molecular standard marker (5 μL, Thermo Fisher Scientific Co., Ltd., Shanghai, China) were loaded onto every precast gel lane (GenScript, 12% polyacrylamide, 15 wells). A MiniProtean electrophoresis apparatus (Bio-Rad Laboratories, Hercules, CA, USA) was used at 4 °C with the following voltage: 80 V for 20 min and 100 V for 80 min. Thereafter, the gel was stained for 40 min and decolored for 10 h by hand using a staining solution and decolorizing liquid (GenScript Biotech Corp, Nanjing, China). The gels were scanned using a molecular imaging system (Gel Doc XR+, Bio-Rad Laboratories, Hercules, CA, USA).

### 2.9. Histology Analysis

Raw WB cubes were cut into small squares with a side length of 5 mm and immediately fixed with 3% glutaraldehyde at room temperature. After the paraffin sections were made, they were dewaxed, stained with Masson and dehydrated. The images were obtained through a microscope (Scope.A1, Carl Zeiss, Gottingen, Germany). 

### 2.10. Data Analysis

15 breasts were used per indicator, and each breast was measured 3 times unless specified. The results are expressed as averages ± standard deviations. Statistical analysis was conducted using SAS software with one-way ANOVA and Duncan’s multiple range tests (SAS Institute Inc., Cary, NC, USA). A *p* value of <0.05 was considered statistically significant. Stress relaxation and creep data were analyzed by nonlinear fitting analysis using Matlab R2016b software (MathWorks, Inc., Natick, MA, USA).

## 3. Results and Discussion

### 3.1. Effect of Different Ultrasound Power on Stress Relaxation of WB 

Some combination models composed of spring and dashpot could describe the viscoelastic properties of WB. The spring represents elasticity, and dashpots are considered to be pure viscosity. In this study, the stress relaxation behavior was described using five-parameter Maxwell model [35]. The modulus σ(t) (Pa) was calculated by the equation below:

(1) $$\sigma \left(t\right)=\varepsilon_{0}\left[E_{0}+E_{1}\exp\left(\frac{-t}{\tau_{1}}\right)+E_{2}\exp\left(\frac{-t}{\tau_{2}}\right)\right],$$

where ε_0_ is the initial strain (30%), *E*_0_ (Pa) is the equilibrium elastic modulus, *E*_1_ (Pa) and *E*_2_ (Pa) are elastic modulus for each Maxwell component, *τ*_1_ (s) and *τ*_2_ (s) are relaxation times, and *E*_0_, *E*_1_, *E*_2_, *τ*_1_, and *τ*_2_ were obtained from the fitting equation according to the experimental stress relaxation data.

**Figure 2:**
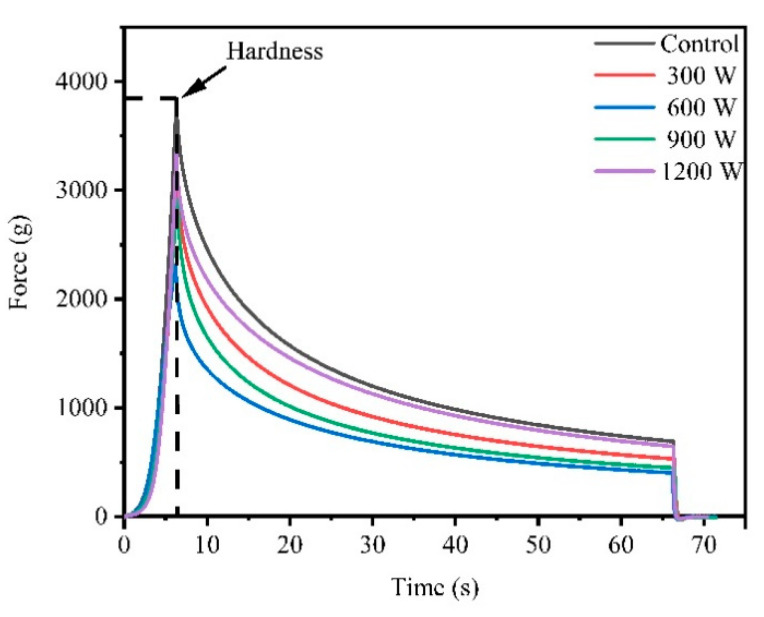
Effect of different ultrasound power on stress relaxation of wooden breast (WB).

The stress relaxation diagram is shown in Figure 2. Force was applied to the meat. When the deformation reached 30% and the deformation stopped, the stress was the maximum value (hardness). With deformation maintained, the stress decreased with time and eventually flattened out, which was a typical feature of stress relaxation.

The hardness values of WB in different treatment groups are shown in Table 1, and the trend of hardness was similar to the trend of shear force as mentioned previously. The Control sample had the highest hardness value (3625.61 g), the 600 W treatment had the lowest hardness (2643.64 g), and the difference between 600 and 900 W was not significant (*p* > 0.05). Dolatowski et al. [36] confirmed that ultrasound had a positive effect on the meat texture and could destroy the integrity of the muscle cells because of its mechanical effect. In addition, ultrasound would damage the muscle fiber structure and release endogenous protease from muscle fibers and eventually enhance tenderness [37].

Table 1 shows the stress relaxation parameters of WB under different treatments obtained after the fitting calculation of the five-parameter Maxwell model. The deformation difficulty of a material is reflected by the elastic modulus. A larger elastic modulus is less likely to deform [38]. The *E*_0_, *E*_1_ and *E*_2_ of 600 W were significantly lower than those of the Control and the 1200 W treatment (*p* < 0.05), indicating that 600 W ultrasonic treatment improved the elasticity and 1200 W decreased the elasticity of WB. The change in the elastic modulus of WB was a comprehensive result of the changes in the fiber structure and other factors on its elastic deformation. The relaxation time was the result of the combined effect of elastic behavior and viscous behavior and was closely related to the binding force between the myofibrillar protein molecules. The longer the relaxation time, the more resistant to deformation [38], the greater the binding force between myofibrillar protein molecules, and the longer the sliding time between them. The *τ*_1_ of 600 W was significantly lower than those of the other groups, indicating the lower strength and better elasticity. This result may be because the protein was denatured, the binding force between myofibrillar protein molecules became smaller, and the relaxation time became shorter. After 600 W, elasticity gradually decreased; that may be caused by the heating effect on the meat surface. 

### 3.2. Effect of Different Ultrasound Power on Creep of WB

**Figure 3:**
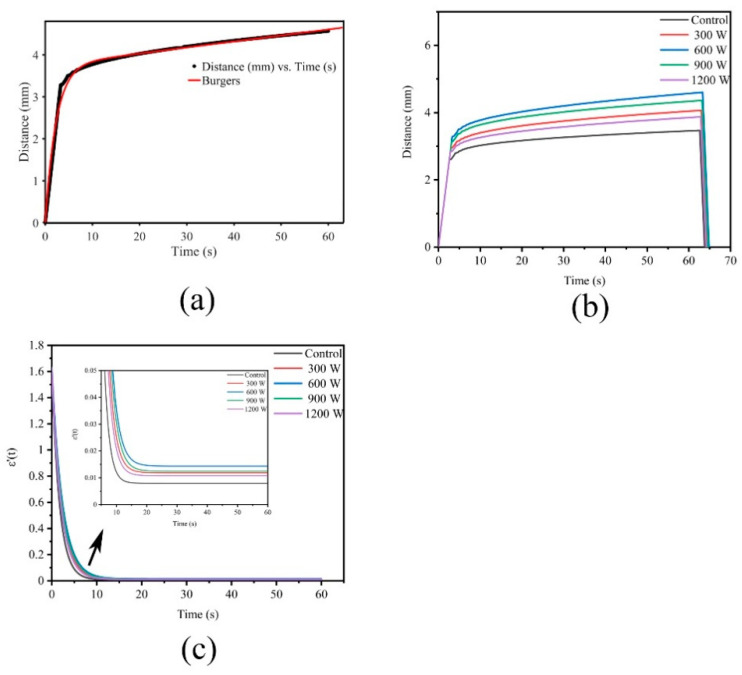
(**a**): Creep fitting; (**b**): Effect of different ultrasound power on creep of WB; (**c**): Effect of different ultrasound power on ε′(t) of WB.

The elastic and viscous parameters can be obtained by modeling, analyzing and fitting the creep process to understand the change in samples. The Maxwell model consists of a spring and a dashpot in series, and the Kelvin model comprises a spring and a dashpot in parallel. However, a certain gap exists between the two models and the actual viscoelastic body. Thus, the Burgers model, which consists of Maxwell and Kelvin units, is widely used in food [39]. Figure 3a showed the Burgers model creep fitting diagram. The total strain ε (*t*) (mm) of the Burgers model was calculated using the following equation:

(2) $$\varepsilon \left(t\right)=\frac{\sigma_{0}}{E_{1}}+\frac{\sigma_{0}}{E_{2}}\left[1-\exp\left(\frac{-t\times E_{2}}{\eta_{2}}\right)\right]+\frac{\sigma_{0}}{\eta_{1}}t,$$

where *E*_1_ (Pa) is the instantaneous elastic modulus of the Maxwell spring, *E*_2_ (Pa) is the retarded elastic modulus of Kelvin, *η*_1_ (Pa·s) is the residual viscosity of dashpot in Maxwell unit, *η*_2_ (Pa·s) is the internal viscosity of dashpot associated with Kelvin, and *σ*_0_ (Pa) is the applied compression stress. *E*_1_, *E*_2_, *η*_1_, and *η*_2_ were obtained from the fitting equation according to the experimental creep data.

Figure 3b shows the creep diagram. The strain applied to meat with a force of 200 g continued to increase with time, and the deformation increased rapidly at the beginning; with the extension of time, the growth rate slowed down and finally stabilized. Wang [40] proposed creep strain as an indicator that describes the rigidity of a material. The softer material with weaker resistance to deformation has a greater creep strain than the stronger one. Among the five groups of curves, the 600 W group exhibited the greatest creep strain and the smallest resistance. 

*E*_1_ reflects the strength and *E*_2_ indicates the resistance to deformation caused by the internal structure of material [39]. As presented in Table 2, *E*_1_ and *E*_2_ gradually decreased, indicating that the meat structure was weakened as the power increased from Control to 600 W. This result may be attributed to the destruction of the muscle fibers and the release of tenderizing enzymes. However, *E*_1_ increased after 600 W, revealing that the meat structure was reinforced. This phenomenon could be caused by heat, which strengthens the structure of meat or enhances intermolecular bonding in MP. The retardation time (*η*_2_/*E*_2_) of Kelvin represents a response of viscoelastic material to the instantaneous application of constant stress [39]. The shorter the retardation time, the faster the elastic response [41]. The retardation time of treatments was significantly higher than that of the Control (*p* < 0.05), indicating that the Control had a faster elastic response than the treatments. 

In poultry plants, the hardness of WB is usually determined by the touch of the experienced grader’s finger. How does the brain interpret the feedback of hardness when the finger presses on the meat? This present work assumed that this phenomenon is related to the change in the speed of pressing the meat. The faster the speed, the softer the meat. In the image, the speed is the slope, and the slope can be obtained by deriving the function. The slope ε′(t) formula is as follows:

(3) $$\varepsilon^{\prime }\left(t\right)=\frac{\sigma_{0}}{E_{2}}\left[\exp\left(\frac{-t\times E_{2}}{\eta_{2}}\right)\right]\left(\frac{E_{2}}{\eta_{2}}\right)+\frac{\sigma_{0}}{\eta_{1}},$$

Figure 3c shows the ε′(t) diagram. The slope decreased rapidly with time from approximately 1.6 at the beginning, revealing that the deformation rate became smaller. Then, the rate of decrease slowed down between 10–20 s. The slope of the Control first started to become gentle, and 600 W was the last. After 20 s, the slope of the 600 W group was maintained at 0.015, which was the highest among all groups, while that of the Control was the lowest. The slope of the treatment was always higher than that of the Control, indicating that the treatment group was softer, easier to deform and corresponded to the result of hardness.

Given that the creep recovery stage was a straight line without any characteristic points, the recovery phase was not explored.

### 3.3. Effect of Different Ultrasound Power on Shear Force of WB

**Figure 4:**
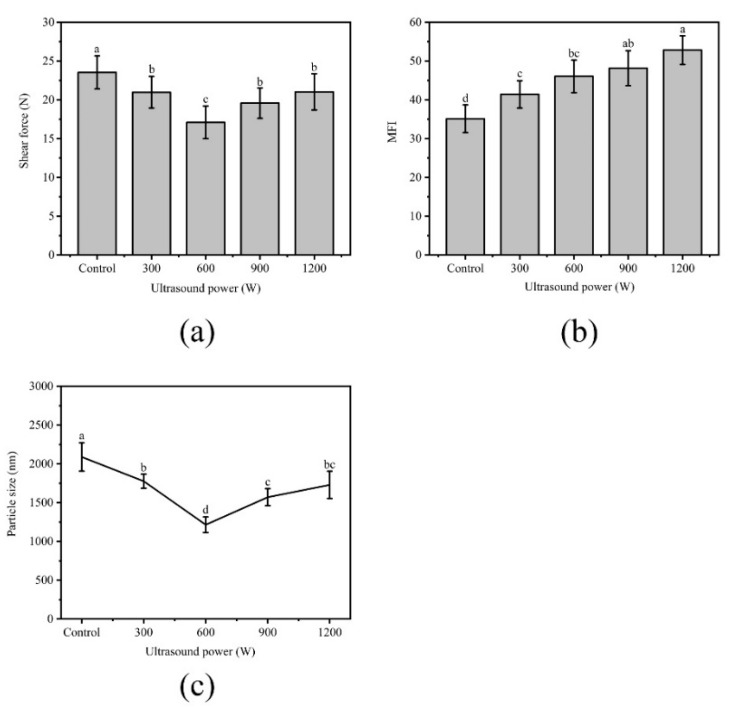
(**a**): Effect of different ultrasound power on shear force of WB; (**b**): Effect of different ultrasound power on the myofibril fragmentation index (MFI) of WB; (**c**): Effect of different ultrasound power on particle size of WB. Error bars are standard deviations (n = 5). Different letters above columns represent a significant difference at *p* < 0.05.

Shear force measurement is the basic indicator of meat tenderness. As shown in Figure 4a, ultrasound had significant (*p* < 0.05) effects on the WB tenderness, which was proven by the low forces under ultrasound. Previous findings [14,42] suggest that the shear force of NB is approximately 19–23% lower than that of WB. Compared with Control, the shear force of the sample significantly decreased from 23.53 N to 17.09 N (*p* < 0.05) under 600 W and decreased by 27%. This result meant that ultrasound can reduce the shear force of WB to a level close to or lower than that of NB. A similar report was published by Alarcon-Rojo [2], who proposed that ultrasound could reduce the shear force of meat. The mechanical effect produced by ultrasound that caused the physical destruction of myofibrillar protein may be the main reason. In addition, the cavitation damaged the cell, thus releasing tenderizing enzymes and enhancing enzyme activity. The promoting effect of salt-soluble protein enrichment from the meat center to the surface may be also responsible, and this protein transfer was considered as a key role in meat tenderization [43]. However, as the shear force increased to 900 W and 1200 W, the heat generated simultaneously with the effect of ultrasound might have a cooking effect on the meat, causing protein denaturation and shrinkage [44,45].

### 3.4. Effect of Different Ultrasound Power on MFI of WB

The MFI value of meat represents the integrity of myofibril and reflects meat tenderness [46]. The high MFI value indicated the structure of myofibril was destroyed severely, which was related to the improved tenderness. As shown in Figure 4b, the MFI values significantly increased from 35.10 in the Control to 52.80 in the 1200 W treatment (*p* < 0.05). This phenomenon could be expounded by cavitation and mechanical action of ultrasonic waves [3], causing powerful damage to the muscle fiber protein during the process.

### 3.5. Effect of Different Ultrasound Power on Particle Size of WB

The decrease in the particle size of the myofibril contributes to the reduction of shear force [47]. The values of particle size from treatments were significantly smaller than those of the Control (*p* < 0.05) as shown in Figure 4c. The smallest average particle size was 1215.00 observed in 600 W (*p* < 0.05), and the mean particle increased to 1728.60 at 1200 W (*p* < 0.05). Changes in particle size are mainly caused by cross-linking and aggregation within the protein molecule [48]. The significant decrease in particle size might be due to the cavitation effect and the physical effect caused by ultrasound that interrupted the noncovalent bonds between MPs. The increase in particle size might be due to the formation of aggregates. The larger the ultrasonic power, the more the hydrophobic groups were exposed, and the hydrophobic interactions between the molecules cause the protein to polymerize, thereby increasing the particle size [49].

### 3.6. Effect of Different Ultrasound Power on Collagen Content of WB

**Figure 5:**
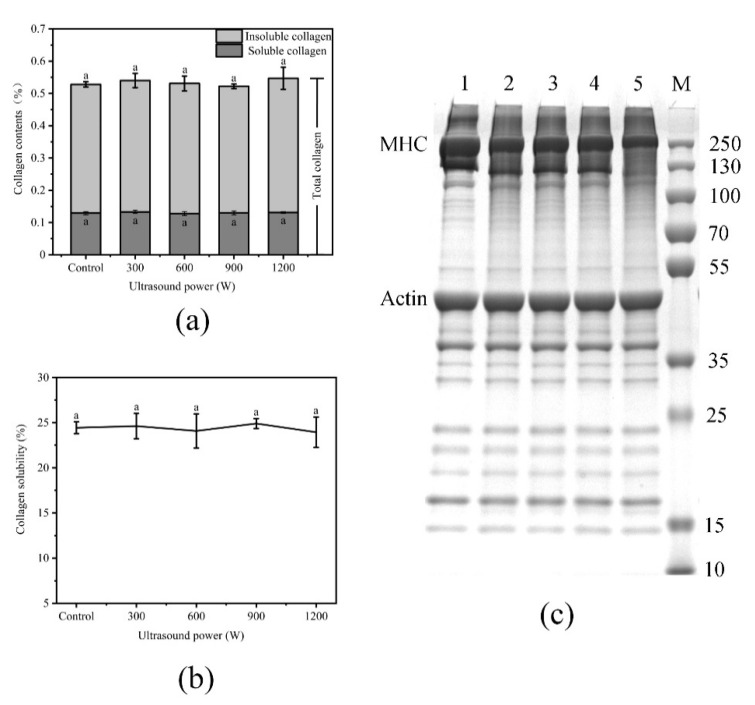
(**a**): Effect of different ultrasound power on collagen content of WB; (**b**): Effect of different ultrasound power on collagen solubility of WB; (**c**): Effect of different ultrasound power on Sodium Dodecyl Sulfate-Polyacrylamide Gel Electrophoresis (SDS-PAGE) of WB, 1–5: Control, 300 W, 600 W, 900 W, 1200 W; M: Marker. Error bars are standard deviations (n = 5). Different letters above columns represent a significant difference at *p* < 0.05.

The effects of ultrasound power on the collagen content and collagen solubility of WB are shown in Figure 5a,b. Collagen content is closely related to the tenderness of meat [50]: the greater the collagen solubility, the more tender the meat [51]. The results of collagen test were similar to those of Lyng [52,53]: ultrasound had no significant effect on collagen content and solubility. The reason may be that there are more connective tissues in WB than in NB, so the intensity of ultrasound cannot effectively destroy collagen. Another reason may be that lipid infiltration in WB hindered the transmission of enough energy, which did not change collagen content and solubility.

### 3.7. Effect of Different Ultrasound Power on SDS-PAGE of WB

The effects of ultrasound power on SDS-PAGE of WB are shown in Figure 5c. The use of ultrasound treatment mainly decreased the intensity of the myosin heavy chains (lanes 1, 2, 3, 4 and 5) and a new band appeared at approximately 15 kDa. With the increase in ultrasonic intensity, the damage to MHC also increased, which indicated that the temperature and pressure created by ultrasound caused the proteins to unfold, leading to the degradation of myofibril protein. The decrease in shear force was related to the degradation of myofibril structure, which indicated that the tenderness of the meat was improved. Rawdkuen et al. [54] found that the degradation of myosin plays an important role in the tenderization of meat. Sawdy et al. [55] also confirmed that the degree of fragmentation of myosin heavy chain is highly correlated with meat tenderness. The more fragments formed by hydrolysis, the better the meat tenderness. However, the heating effect caused the evaporation of water and the shrinkage of the structure [56], which decreased the tenderness at 900 W and 1200 W.

### 3.8. Effect of Different Ultrasound Power on Histology of WB

**Figure 6:**
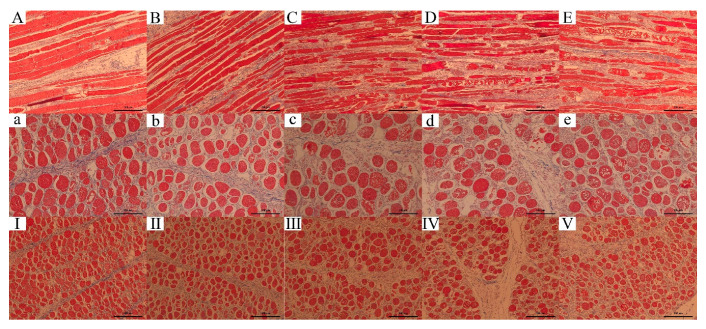
Effect of different ultrasound power on histology of WB. Masson staining of tissue. From left to right are the Control, 300 W, 600 W, 900 W and 1200 W groups. (**A**–**E**): longitudinal section of myofibril, scale bar = 200 μm; (**a**–**e**): transverse section of myofibril, scale bar = 100 μm; (**I**–**V**): transverse section of myofibril, scale bar = 200 μm.

Histological observations (Figure 6) show some typical characteristics of WB, including that round, variably sized fibers were separated from each other or replaced by connective tissue [57] and necrotic fibers [58,59].

In the longitudinal section of the myofibril (A–E), the untreated WB fibers were thick and relatively intact. After treatments, the complete fibers were broken into small pieces, and the fiber diameter decreased. With the enhancement of ultrasonic power, more cavities were observed and more damaged fiber with increased space also appeared. In the transverse section of the myofibril (a–e), similar results were found. In the Control, the fibers were relatively close and intact. As the ultrasound power increased, it also showed the space and cavities increased. The fiber cell membrane was broken, and the tissue content was released. Cavitation effect caused muscle fibers to break along the Z line [5], enhanced the activity of enzymes [56], promoted the destruction of the fiber structure, and resulted in the tenderization effect.

The connective tissue was not damaged as indicated in histology analysis (I–V). However, in the Figure 6E, the connective tissue showed shrinkage and curved because of heat [60], indicating the tenderness decreased. Another point that must be taken into account was the enzyme denaturation and protein oxidation, which can increase toughness [5,61].

## 4. Conclusions

Stress relaxation and creep showed that the hardness of the WB treated with 600 W ultrasound decreased and its elasticity increased. Analysis of shear force indicated that the 600 W ultrasonic power can significantly tenderize WB as shown by the results of the decreased shear force valve, as well as increased MFI and, decreased particle size. However, the meat became hard at treatments higher than 600 W because the heating effect caused water loss, connective shrinkage and even the protein oxidation. In a word, stress relaxation and creep not only predicted texture change of WB after ultrasound, but also had the potential to be applied in meat quality analysis. The 600 W ultrasound treatment was an effective strategy to reduce economic losses of WB.

## Figures and Tables

**Table 1:** Effects of different ultrasound power on stress relaxation parameters of WB.

	Ultrasound Power				
	Control	300 W	600 W	900 W	1200 W
Hardness (g)	3625.61 ± 148.39 ^a^	3119.98 ± 90.70 ^bc^	2643.64 ± 208.30 ^d^	2901.10 ± 100.08 ^cd^	3383.16 ± 374.68 ^ab^
*E*_0_ (×10 Pa)	3754.58 ± 262.60 ^a^	3213.62 ± 308.86 ^bc^	2860.03 ± 4130.2 ^cd^	2523.34 ± 419.06 ^d^	3505.26 ± 260.89 ^ab^
*E*_1_ (×10 Pa)	6618.14 ± 417.43 ^a^	5817.28 ± 120.03 ^ab^	5211.64 ± 887.22 ^b^	6143.82 ± 436.91 ^ab^	6320.61 ± 1322.57 ^a^
*τ*_1_ (s)	2.49 ± 0.15 ^a^	2.20 ± 0.16 ^b^	1.71 ± 0.24 ^c^	2.23 ± 0.16 ^b^	2.30 ± 0.18 ^ab^
*E*_2_ (×10^2^ Pa)	1160.56 ± 68.74 ^a^	964.67 ± 37.58 ^c^	776.16 ± 59.76 ^d^	865.42 ± 31.66 ^d^	1061.38 ± 114.05 ^b^
*τ*_2_ (s)	21.10 ± 0.69 ^a^	20.15 ± 0.94 ^a^	19.97 ± 1.50 ^a^	18.31 ± 0.49 ^b^	20.17 ± 1.48 ^a^
R^2^	0.9992	0.9990	0.9985	0.9989	0.9991

Data presented are means and standard deviations from five times. Different letters represent a significant difference at *p* < 0.05.

**Table 2:** Effects of different ultrasound power on creep parameters of WB.

	Ultrasound Power				
	Control	300 W	600 W	900 W	1200 W
*E*_1_ (×10^5^ Pa)	3618.20 ± 186.34 ^a^	2581.80 ± 110.64 ^c^	1776.00 ± 129.69 ^e^	2373.80 ± 101.95 ^d^	3051.20 ± 149.95 ^b^
*η*_1_ (×10^2^ Pa·s)	4972.80 ± 160.78 ^a^	3399.60 ± 128.01 ^c^	2702.20 ± 160.45 ^e^	2959.00 ± 181.04 ^d^	3792.00 ± 228.27 ^b^
*E*_2_ (Pa)	1389.20 ± 74.36 ^a^	1155.80 ± 71.36 ^b^	1118.40 ± 55.15 ^b^	1194.00 ± 112.06 ^b^	1191.20 ± 33.18 ^b^
*η*_2_ (Pa·s)	2414.00 ± 14.40 ^c^	2434.60 ± 19.77 ^abc^	2463.80 ± 39.33 ^ab^	2476.80 ± 52.30 ^a^	2426.20 ± 26.88 ^bc^
*η*_2_/*E*_2_ (s)	1.74 ± 0.10 ^b^	2.11 ± 0.15 ^a^	2.21 ± 0.10 ^a^	2.09 ± 0.17 ^a^	2.04 ± 0.06 ^a^
R^2^	0.9868	0.9881	0.9893	0.9891	0.9877

Data presented are means and standard deviations from five times. Different letters represent a significant difference at *p* < 0.05.

## Data Availability

Data sharing is not applicable to this article.
